# Thermal Conductivity Characteristics and Prediction Model of Silty Clay Based on Actively Heated Fiber-Optic FBG Method

**DOI:** 10.3390/s25175393

**Published:** 2025-09-01

**Authors:** Shijun Hu, Honglei Sun, Miaojun Sun, Guochao Lou, Mengfen Shen

**Affiliations:** 1 College of Civil Engineering, Zhejiang University of Technology, Hangzhou 310023, China; hushijun13@mails.ucas.edu.cn (S.H.); sunhonglei@zju.edu.cn (H.S.); 2 POWERCHINA Huadong Engineering Corporation Limited, Hangzhou 310014, China; sun_mj2@hdec.com; 3 Zhejiang Engineering Research Center of Marine Geotechnical Investigation Technology and Equipment, Zhejiang Huadong Construction Engineering Corporation Limited, Hangzhou 311122, China; 4 Zhejiang Key Laboratory of Green Construction and Intelligent Operation & Maintenance for Coastal Infrastructure, Zhejiang University of Technology, Hangzhou 310014, China; 5 Zhejiang Sanjian Construction Group Co., Ltd., Hangzhou 310016, China; louguochao316@163.com

**Keywords:** soil thermal conductivity, actively heated fiber-optic method (AHFO), fiber Bragg grating (FBG), thermal response, prediction model

## Abstract

Soil thermal conductivity (*λ*) is a critical parameter governing heat transfer in geothermal exploitation, nuclear waste disposal, and landfill engineering. This study explores the thermal conductivity characteristics of silty clay and develops a prediction model using the actively heated fiber-optic method based on fiber Bragg grating technology. Tests analyze the effects of particle content (silt and sand), dry density, moisture content, organic matter (sodium humate and potassium humate), and salt content on *λ*. Results show *λ* decreases with increasing silt, sand, and organic matter content, while it increases exponentially with dry density. The critical moisture content is 50%, beyond which *λ* declines, and *λ* first rises then falls with salt content exceeding 2%. Sensitivity analysis reveals dry density is the most influential factor, followed by sodium humate and silt content. A modified Johansen model, incorporating shape factors correlated with influencing variables, improves prediction accuracy. The root mean squared error decreases to 0.087, and coefficient of determination increases to 0.866. The study provides an accurate method for measuring thermal conductivity and enhances understanding of the heat-transfer mechanism in silty clay.

## 1. Introduction

Soil thermal conductivity (*λ*) is a key physical parameter characterizing the heat conduction capacity of soil, directly governing the temperature field distribution and heat-transfer efficiency during soil heat-transfer processes [1]. Investigation into the influencing factors and intrinsic mechanisms of soil thermal conductivity holds significant theoretical and practical value for heat-transfer analysis, model development, and optimal design in thermal engineering projects, such as the exploitation of deep mineral and geothermal resources [2,3], the regulation of heat conduction in deep geological disposal of nuclear waste [4,5,6], the design for thermal stability of landfill liner systems [7], etc.

Extensive research exists on soil thermal conductivity testing, with the thermal probe method and thermal response test (TRT) being most widely applied. The thermal probe method, an accurate point-measurement technique, uses a heated sensor-equipped metal probe inserted into soil [8,9] but reflects only local thermal properties due to limited heating range from sensor length constraints. The TRT, the most common engineering method, employs line or cylindrical heat source models for data inversion to characterize average thermal conductivity of larger soil volumes around boreholes [9,10,11], yet traditional TRTs suffer from long duration and complex data processing. With the development of optical fiber sensing technology, actively heated fiber-optic (AHFO) methods, such as fiber Bragg grating (AHFO-FBG) and distributed temperature sensing (AHFO-DTS) technologies, have achieved rapid development by enabling point or distributed continuous measurement of soil thermal conductivity through optical cables implanted in soil [12,13,14,15,16,17]. The AHFO method uses an actively heated optical cable as the heat source, monitors changes in the soil’s temperature distribution along the optical fiber, and calculates the soil’s thermal conductivity via the line heat source model—similar to the widely used thermal probe method. Both share the core principle: analyzing the temperature response of an embedded heated “hot wire” to determine the material’s heat conduction efficiency. Hainar et al. [15] proposed an AHFO-DTS measurement method for the effective thermal conductivity of multi-layered soils through indoor thermal response model tests. Compared with traditional thermal response tests, this method has significant advantages such as shorter time consumption, simpler installation, and uniform heating. Zhang et al. [16] compared conventional thermal response test results with those from a distributed AHFO-DTS test in the same borehole, confirming AHFO-DTS reliability with differences less than 5%. This novel method also consumes significantly less energy and reduces test time considerably. Cheng et al. [17] applied optical frequency domain reflectometry to investigate the influence of various factors on thermal conductivity measurements and compared the results with those obtained via the thermal probe method. It was found that the fiber-optic-based method for soil thermal conductivity measurement exhibits high accuracy. In addition, AHFO-FBG has also been applied to measure parameters such as moisture content, ice content, and dry density of geotechnical materials [18,19,20,21,22]. The above studies show the AHFO method performs excellently in temperature measurement, and its application to soil thermal conductivity measurement is expected to yield favorable results.

With soil thermal conductivity testing, scholars found that numerous factors influence the thermal conductivity of geotechnical materials, mainly including mineral composition, particle size and gradation, water content, porosity, saturation, salt content, organic matter, and temperature, among others [23,24,25]. Abu-Hamdeh et al. [26] found that the thermal conductivity of sand, clay, sandy loam, and loam increased by increasing water content and dry density, and the thermal conductivity of clay loam was lower than that of sand. Lee et al. [27] studied the effects of dry density, water content, and temperature on the thermal conductivity of bentonite and found that λ increased with the increase in water content and dry density, and the influence of temperature on λ could be ignored. Zhao et al. [28] measured thermal properties of six peat soils and proposed a new model accounting for porosity, saturation, organic matter content, and texture effects on thermal conductivity, showing potential for numerical algorithms describing coupled heat-mass transfer in multiphase soils. Lu et al. [29] established the parallel-series mixed model by the series–parallel combination of various components of porous media and verified the accuracy of the model prediction. However, research on the thermal conductivity of silty clay remains limited, and existing soil thermal conductivity prediction models suffer from certain limitations due to their strong localization constraints.

In this study, the effects of soil particle composition, water content, dry density, organic matter content, and salt content on the thermal conductivity of silty clay were systematically investigated using the AHFO-FBG technique. The heat-transfer mechanism was discussed, and a modified λ prediction model was developed. This research contributes to a better understanding of the thermal conductivity characteristics of silty clay. The accurate thermal conductivity prediction model can provide reliable thermal design parameters for geothermal exploitation, nuclear waste disposal, and other related thermal engineering projects, which is conducive to optimizing engineering design and improving project safety and efficiency.

## 2. Principles of the AHFO-FBG Method

Fiber Bragg grating (FBG) takes advantage of the natural UV photosensitivity of fiber. When the UV laser irradiates the fiber core, the light with a specific wavelength cannot pass through, and the refractive index of the irradiated fiber core will change periodically or irregularly along the fiber axis, forming phase grating [30,31]. The specific wavelength is called the central wavelength *λ*_B_, which satisfies the following relation:

*λ*_B_ = 2*n*_eff_*Λ* (1)

where *n*_eff_ is the effective refractive index (dimensionless), and *Λ* is the spatial period of the FBG (nm).

The *λ*_B_ drifts due to the variations of external strain and temperature [32] as follows:

(2) $$\frac{\Delta \lambda_{B}}{\lambda_{B}}=(\alpha +\xi )\Delta T+(1-P_{e})\Delta \varepsilon$$

where *α* is the thermal expansion coefficient of fiber; *ξ* is the thermo-optical coefficient of fiber; Δ*T* is the temperature variation; *P*_e_ is the elastic-optical coefficient of fiber; and Δ*ε* is the axial strain of fiber.

In this study, since the sensor was in a static state during the test and was not affected by any external force, the effect of strain on wavelength could be ignored. Meanwhile, due to the long heating time, it could be considered that the change in wavelength in the test was caused only by temperature. Therefore, Equation (2) could be further simplified as:

(3) $$\frac{\Delta \lambda_{B}}{\lambda_{B}}=\left(\alpha +\xi \right)\Delta T$$

An alundum tube with an internal heating function was used to encapsulate FBG and resistance, which can be used as a thermal probe sensor. The FBG alundum tube sensor can be approximately regarded as an infinite cylindrical heat source [33]. Assuming the soil is a homogeneous isotropic medium heating under a line power with the constant electrical current *Q* (W/m), the analytical solution for temperature according to the line heat source model is as follows [13,14,15,16,34]:

(4) $$\Delta T=T\left(t\right)-T_{0}=\frac{Q}{4\pi \lambda }\left[\ln(t)+4\pi R\lambda +\ln\left(\frac{4K}{a^{2}c}\right)\right]\;t>>\frac{a^{2}}{K}$$

where Δ*T* is the temperature rise (°C); *T*(*t*) is the temperature of the heat source (°C) corresponding to the heating time *t*; *T*_0_ is the initial ambient temperature (°C); *λ* is the thermal conductivity of soil (W∙m^−1^∙K^−1^); *R* is the thermal resistance per unit length (K^−1^∙W^−1^∙m^−1^) between the sensor and the soil wall; *K* is the thermal diffusivity (m^2^∙s^−1^) of the soil; *a* is the outer diameter of the sensor (m); *c* is a constant equals to exp(*γ*) = 1.781; and *γ* is the Euler’s constant (=0.5772).

According to the definitions of Equation (4), only time *t* is a variable, and all the other terms are constants, which means the Δ*T* increases with ln(*t*) linearly. So, Equation (4) can be rewritten as:

(5) ΔT=kln(t)+b

where *k* is the slope of the linear relationship between Δ*T* and ln(*t*), which equals to *Q*/(4π*λ*); *b* is the intercept of the line.

Then the thermal conductivity of soil *λ* can be determined as follows:

(6) $$\lambda =\frac{Q}{4\pi k}$$

## 3. Materials and Methods

### 3.1. Tested Soil

**Figure 1:**
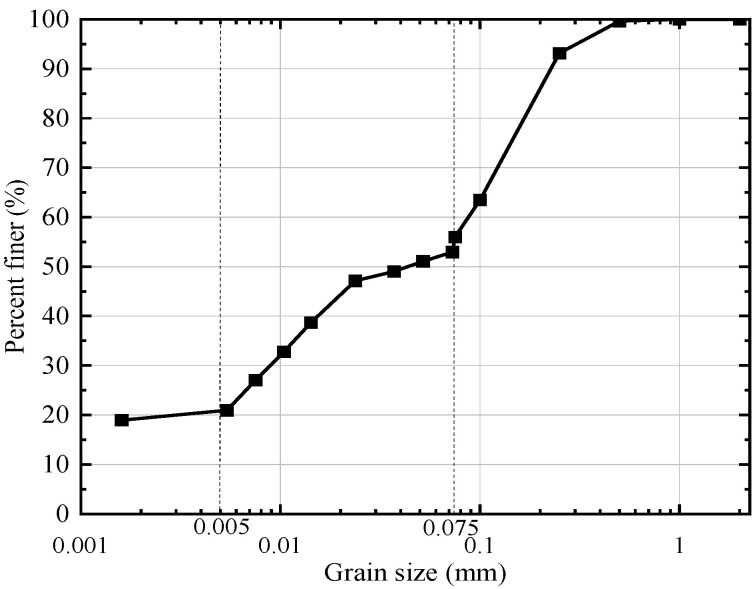
Grain size distribution of test soil.

The soil used in this study was sampled from Taizhou City, Zhejiang Province, China. The properties of the tested soil are listed in Table 1, and its grain size distribution is shown in Figure 1. The maximum dry density and optimum moisture content were determined via the standard Proctor compaction test. The organic matter content was measured using a muffle furnace. According to the Unified Soil Classification System (USCS), the tested soil is classified as low plasticity clay (CL).

### 3.2. Test Device

**Figure 2:**
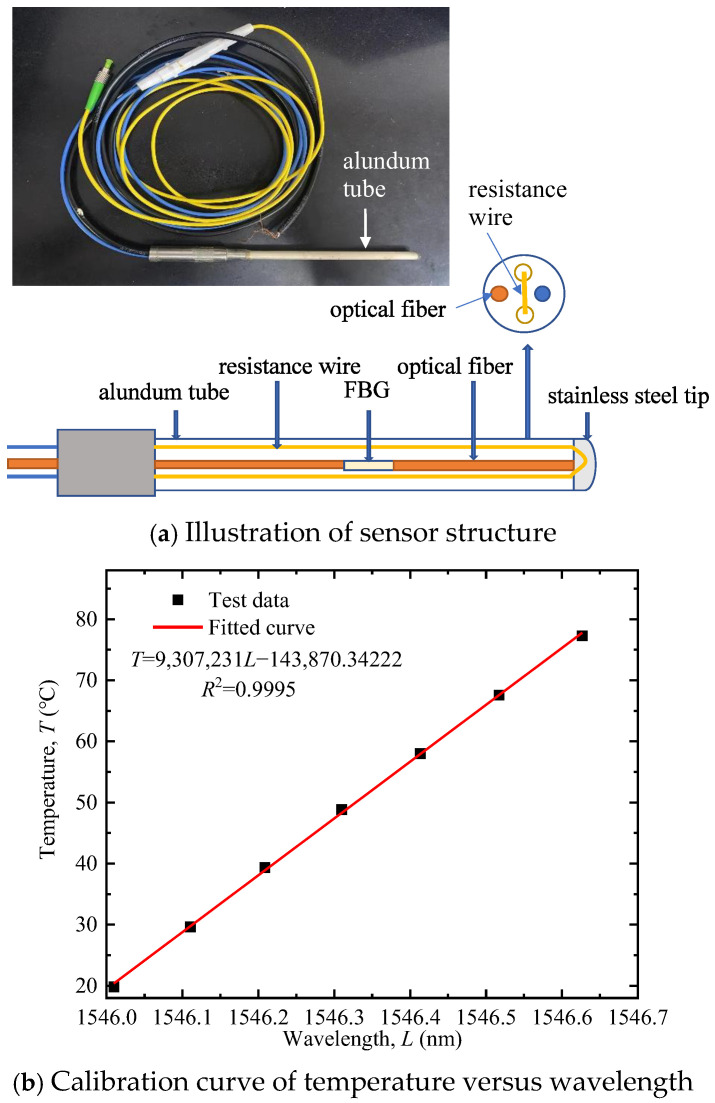
FBG-based alundum tube-packed sensor.

The sensor and demodulator used in this study were manufactured by Suzhou NanZee Sensing Technology Co., Ltd., based in Suzhou, China. The basic structure of the FBG-based alundum tube-packed sensor, illustrated in Figure 2a, comprises a four-hole alundum tube (outer diameter 4.5 mm), a resistance wire, and a FBG. The alundum tube, with high rigidity, strength, excellent insulation, high-temperature resistance, and good thermal conductivity, protects the FBG from shear damage. The FBG fiber is placed in one small hole with one end stress-relaxed to eliminate strain interference on measurement results. The resistance wire runs through two holes in a U-shape and can be electrically heated via a power source. This FBG sensor operates within a temperature range of −40 °C to 80 °C with a measurement accuracy of 1‰ F.S. The wavelength–temperature correlation of the sensor was calibrated before factory shipment (Figure 2b). The calibration curve exhibits a strong linear relationship with a fitting degree of 0.9995, enabling direct conversion of wavelength to temperature with high accuracy.

**Figure 3:**
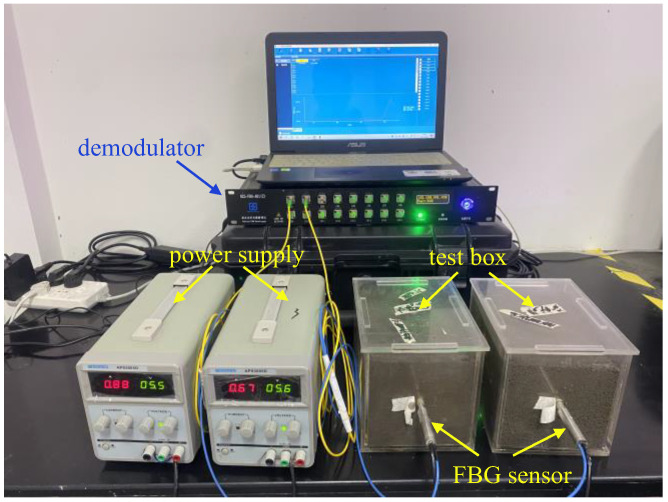
Measurement system.

The measurement system is shown in Figure 3. This system comprises four components: a DC regulated power supply for heating, a test box (20 cm × 15 cm × 15 cm) integrated with a FBG fiber sensor, a FBG fiber demodulator (with technical parameters listed in Table 2), and a computer for data processing. The test box size was verified by placing a FBG sensor at its horizontal edge while the central FBG sensor operated. No wavelength change was observed in the edge sensor throughout the test, confirming the box is sufficiently large to meet the line heat source model assumption.

### 3.3. Experimental Scheme and Procedures

A series of experiments were designed (Table 3) using the controlled variable method to investigate the effects of particle content (silt and sand content), dry density, moisture content, organic matter content, and salt content on soil thermal conductivity. The variation ranges of these variables were determined based on the typical values of the test soil, as suggested by local geotechnical engineers who referenced the survey report results of multiple regional test sites.

**Figure 4:**
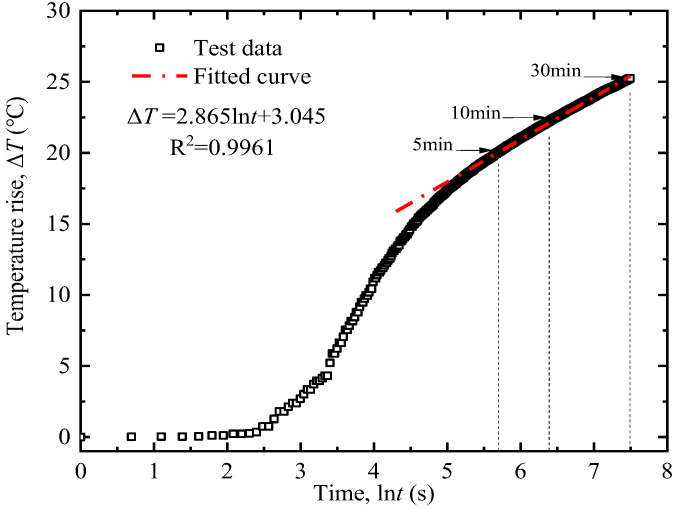
Typical result of temperature versus ln (time) for the test soil.

The measurement errors mainly stem from an unstable power supply, inaccurate fiber calibration, and non-uniform soil samples. To mitigate these errors, the following experimental procedures were implemented:

(1) Soil sample preparation: Based on the experimental scheme in Table 3, calculate the required mass of dry soil and additives. Prepare the soil sample with the target moisture content, and then allow it to maintain for 24 h to ensure uniform water distribution within the soil.
(2) Model sample preparation: Open the lid of the text box (see Figure 3). Calculate the filling height of each soil layer based on the target dry density. Fill the prepared soil into the test box in layers. Roughen the soil surface to minimize layering effects. When the filling reaches the preset height of the corundum tube, embed the corundum tube at the geometric center of the soil box. Then fill the remaining soil in layers, taking care to keep the corundum tube in its original position.
(3) Measurement by the AHFO-FBG method: Activate the demodulator. Measure the room temperature using the FBG sensor and verify it with a thermometer. After confirming the optical fiber is intact, connect the power supply to start heating at a constant power of 20 W/m for 10 min. Wavelength variations during the heating process are converted to temperature rises using the calibration curve in Figure 2b, and the temperature rise-ln (time) curve is plotted to determine its slope *k*. A typical result is shown in Figure 4. Soil thermal conductivity is then calculated via Equation (6). Each test was repeated three times, and the average value was adopted to reduce random errors.
(4) The above steps were repeated to complete all tests listed in Table 3.

## 4. Results

### 4.1. Thermal Conductivity Analysis

**Figure 5:**
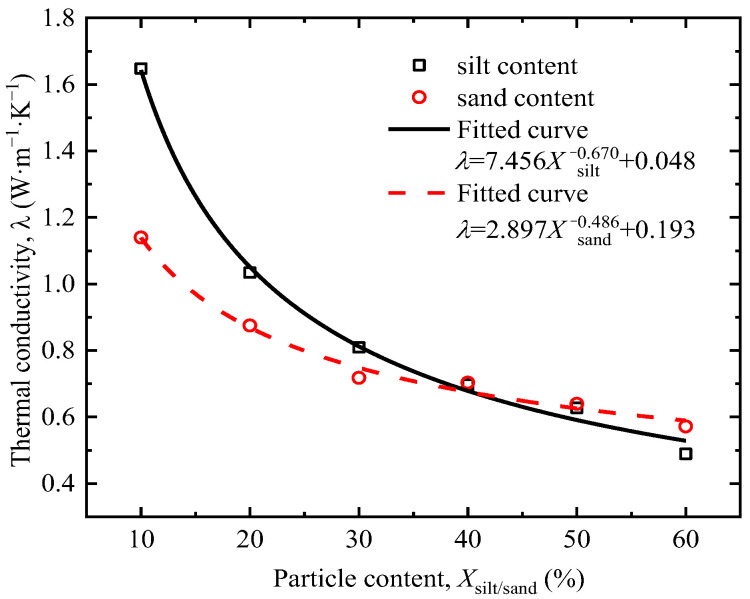
Relationship between particle gradation and thermal conductivity.

Figure 5 shows the thermal conductivity of tested clay at different silt and sand contents. With the increase in sand or silt content, the thermal conductivity of the soil continuously decreases. This is mainly because when the water content of the tested soil is 20%, the increase in sand or silt content leads to an increase in soil porosity, which increases the volume of air. Since the thermal conductivity of gas is lower than that of solids, this results in a decrease in the overall thermal conductivity of the soil. Overall, the thermal conductivity of soil is more influenced by silt content than sand content, primarily because silt, due to its fine particles, close contact, and strong adaptability to water content, generally has higher thermal conductivity than sand.

**Figure 6:**
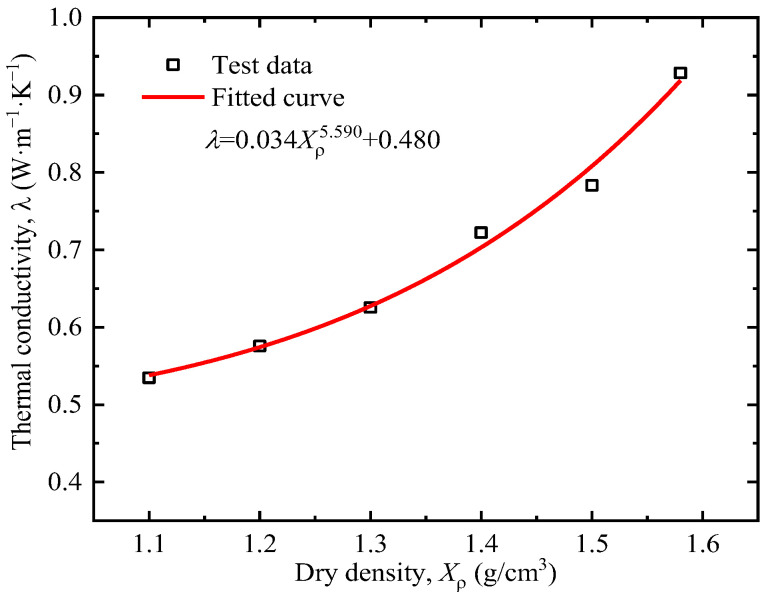
Relationship between dry density and thermal conductivity.

Figure 6 shows the thermal conductivity of tested clay at different dry densities. The thermal conductivity of soil increases with the increase in dry density. The increase in compactness leads to a decrease in the void ratio, a reduction in pore volume (especially large pores), and a relative increase in the proportion of water in the pores. Since the thermal conductivity of most soil minerals is basically above 2.0 W·m^−1^·K^−1^, the thermal conductivity of water ranges from 0.45 to 0.6 W·m^−1^·K^−1^, and the thermal conductivity of air is about 0.024 W·m^−1^·K^−1^ [35]. Therefore, soil with higher compactness has a higher degree of saturation and a greater thermal conductivity.

Moisture content is another important factor affecting the thermal conductivity of soil. In the test, the moisture content of the soil was designed to vary between 10% and 155%. It is worth noting that when the moisture content exceeds the liquid limit of 40%, the soil transitions from a plastic state to a liquid state. Therefore, the dry density of the soil was controlled at 1.3 g/cm^3^ only when the moisture content was low enough for density control.

**Figure 7:**
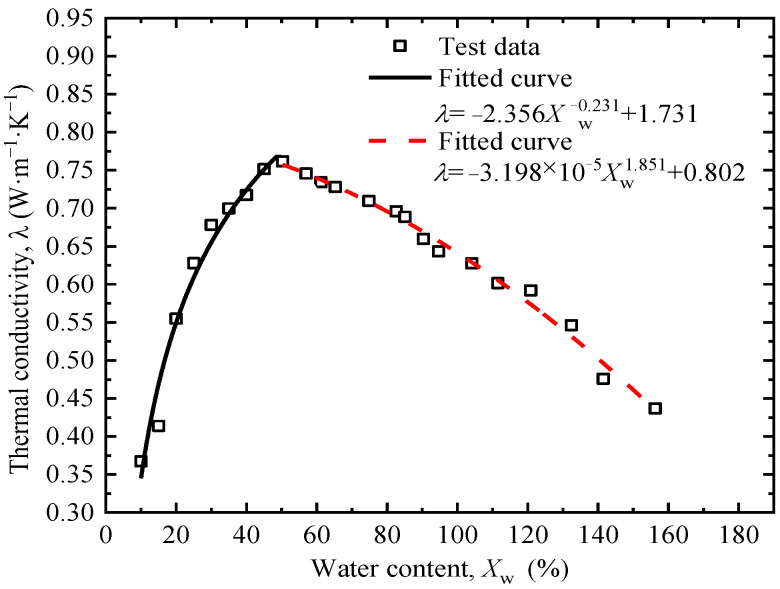
Relationship between moisture content and thermal conductivity.

Figure 7 shows the thermal conductivity of the tested soil first increases and then decreases with the increase in moisture content. The critical moisture content for the trend change is about 50%, which is slightly higher than the liquid limit. When influencing factors such as dry density remain unchanged, soil particles, water, and air all participate in heat conduction when the water content is less than the critical moisture content. With the increase in water content, part of the water forms a water film wrapping soil particles, leading to an increasing number of water bridges between soil particles, which continuously expands the thermal conduction paths of the soil [36]. As the thermal conductivity of water is much higher than that of air, the increase in water content enhances the thermal conductivity of the soil. When the pores of the soil are filled with water, the thermal resistance coefficient of the soil is minimized, and the thermal conductivity is maximized. When the water content exceeds the critical moisture content, heat conduction occurs only within and between the solid and liquid phases. The thermal conduction of the soil is mainly dominated by water, whose thermal conductivity is much lower than that of soil minerals. Then the increase in water content causes the overall thermal conductivity of the soil to gradually decrease.

**Figure 8:**
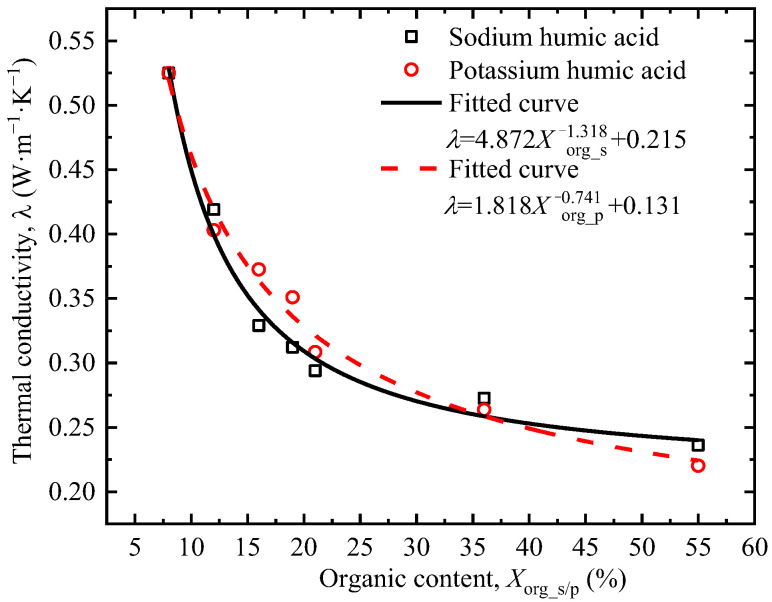
Relationship between the organic matter content and thermal conductivity.

Humic acid accounts for about 50% to 80% of total organic matter in natural soils. The soluble salts of humic acid, potassium, and sodium humates were selected to mimic its native speciation in soil environments. Figure 8 shows that thermal conductivity decreases with the increase in organic matter content. When the organic matter content is low, the increase in organic matter content significantly reduces the thermal conductivity; when the organic matter content is high, the reduction in thermal conductivity is not obvious. Since the thermal conductivity of organic matter is between 0.1 and 0.4 W·m^−1^·K^−1^, which is much lower than that of soil minerals, organic matter fills the spaces between soil particles in a loose and porous form, replacing part of the mineral particles or forming isolation layers, thereby directly reducing the continuity of the thermal conductive matrix of the soil. The increase in potassium humate content reduces the thermal conductivity of soil slightly more than sodium humate. The reasons for this phenomenon need to be confirmed through further experiments.

**Figure 9:**
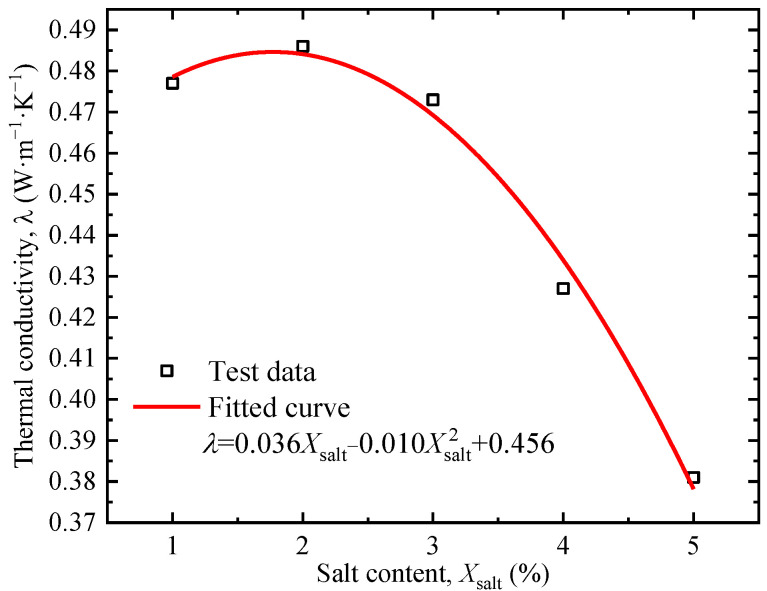
Relationship between salt content and thermal conductivity.

Figure 9 shows the soil thermal conductivity under different salt contents. The thermal conductivity of soil exhibits a two-stage characteristic with the increase in the salt content: when the salt content is lower than 2%, the thermal conductivity increases with the increase in salt content; when it exceeds 2%, the trend is reversed. The trend is consistent with the results of Lyu et al. [37]. The increase in the early stage is due to the increase in Na^+^ and Cl^−^ ion concentrations in the NaCl solution. The negative charge of soil particles adsorbs Na^+^, leading to double-layer compression, the reduction of repulsive force range between particles, and thinning of the public hydration film, thus enhancing the thermal conductivity. The decrease in the later stage is due to two aspects: first, the thermal conductivity of the NaCl solution decreases with the increase in concentration, which leads to the decrease in soil thermal conductivity when the water content remains unchanged; second, the interaction between clay particles and salt ions produces flocculation and aggregation [37,38]. The flocculent aggregates are separated by free water, which greatly reduces the effective contact area between soil particles, and the higher the degree of flocculation, the lower the thermal conductivity.

### 4.2. Sensitivity Analysis of Influencing Factors

To obtain the sensitivity magnitudes of different factors, this study uses the sensitivity factor S*(X*) [39] to reflect the sensitivity of influencing factors. The sensitivity factor is defined as the ratio between the relative error of system characteristic *P* with respect to the reference value X* to the relative error of parameters with respect to the X*, and its calculation formula is shown in Equation (7):

(7) $$S*(X^{*})=\left(\frac{\left|\Delta P\right|}{P}\right)/\left(\frac{\left|\Delta X^{*}\right|}{X^{*}}\right)=\left|\frac{\Delta P}{X^{*}}\right|\frac{X^{*}}{P}$$

When $\left|\Delta X^{*}\right|/X^{*}$ is small, Equation (7) can be approximately expressed as Equation (8):

(8) $$S*\left(X^{*}\right)=\left|{\left(\frac{dP}{dX}\right)}_{X=X^{*}}\right|\frac{X^{*}}{P}$$

S*(X*) is a dimensionless non-negative number, which can be used to compare the sensitivity of various factors. The larger its value, the more sensitive the system characteristic *P* is to the change of parameter *X* under the reference value.

The system characteristic *P*, i.e., thermal conductivity *λ* in this study, was obtained by the fitting results in Figure 5, Figure 6, Figure 7, Figure 8 and Figure 9. The sensitivity factors of the influencing factors, including particle content, dry density, moisture content, organic matter content, and salt content, were determined by Equation (8) and summarized in Table 4.

Subsequently, the sensitivity factors under their reference values were calculated and ranked. The reference values were determined under the initial state of soil. The ranking of sensitivity factors under the reference values (Table 3) is as follows: dry density > sodium humate > silt content > potassium humate > moisture content > sand content > salt content. This indicates that under the reference values of these factors, the thermal conductivity of the soil is most sensitive to the changes in dry density near its reference value and least sensitive to the changes in salt content. If the measured value deviates from the actual value by 20%, the relative error of thermal conductivity caused by the measurement error of dry density can reach 1.313 × 20% = 26.26%, while the relative error caused by salt content is only 0.033 × 20% = 0.66%. In light of this, it is necessary to reduce the measurement error of dry density by increasing the number of repeated tests and to retain as many decimal places as possible during data collection. It is worth noting that the sensitivity ranking is significantly influenced by the selection of reference values. For instance, if the reference value of dry density is set to 1.1, its sensitivity factor will decrease to 0.602. This thus necessitates case-specific investigations.

### 4.3. Model Prediction

The Johansen (1977) model [23] is a cornerstone in soil thermal conductivity prediction, extensively cited in geotechnical, environmental, and energy engineering. The model pioneeringly introduced the normalized thermal conductivity (*λ*_r_, also known as Kersten number) concept by linking thermal conductivity (*λ*) to the soil thermal conductivities under fully saturated (*λ*_sat_) and dry (*λ*_dry_) conditions, as defined in Equation (9):

(9) $$\lambda =(\lambda_{\mathrm{sat}}-\lambda_{\mathrm{dry}})\lambda_{r}+\lambda_{\mathrm{dry}}$$

(10) $$\lambda_{\mathrm{sat}}=\lambda_{w}^{n}\lambda_{s}^{1-n}$$

(11) $$\lambda_{\mathrm{dry}}=\frac{0.137\rho_{d}+64.7}{2650-0.947\rho_{d}}$$

(12) $$\lambda_{r}=\left\{\begin{array}{l} 0.7\log S_{r}+1\;\mathrm{for}\;\mathrm{median}\;\mathrm{and}\;\mathrm{fine}\;\mathrm{sands}\\ \log S_{r}+1\;\mathrm{for}\;\mathrm{fine}\;\mathrm{soils}\\ S_{r}\;\mathrm{for}\;\mathrm{frozen}\;\mathrm{fine}\;\mathrm{sand}\;\mathrm{and}\;\mathrm{fine}\;\mathrm{soils} \end{array}\right.$$

where *λ*_w_ and *λ*_s_ are the thermal conductivities of water and solid, respectively, W∙m^−1^∙K^−1^; *n* is the soil porosity; *r*_d_ is the dry density; and *S*_r_ is the degree of saturation.

While the Johansen (1977) model [23] accounts for dry density and moisture content, it does not adequately consider particle composition, organic matter content, or salt concentration that lead to persistent errors in predicted results. To address this, a novel relationship between *λ*_r_ and *S*_r_ is proposed in Equation (13), integrating previous model frameworks across the full range of water content:

(13) $$\lambda_{r}=\exp(\alpha -S_{r}^{-\beta })$$

where *α* and *β* are the shape factors of the *λ*_r_(*S*_r_) curve that affect the slope and growth rate of the curve. Assume that *α* and *β* are linearly correlated with the particle content, dry density, moisture content, organic matter content, and salt content. The expressions of *α* and *β* were then calibrated with the test data, which are shown in Equations (14) and (15), respectively. As shown, the shape factor *β* is only sensitive to the silt content and dry density:

(14) $$\begin{array}{l} \alpha =-0.033X_{\mathrm{silt}}-0.015X_{\mathrm{sand}}+0.646X_{\rho }-0.001X_{w}\\ \;-0.034X_{\mathrm{org}\_s}-0.032X_{\mathrm{org}\_p}-0.128X_{\mathrm{salt}}+0.63 \end{array}$$

(15) $$\beta =0.620X_{\mathrm{silt}}+0.169X_{\rho }$$

**Figure 10:**
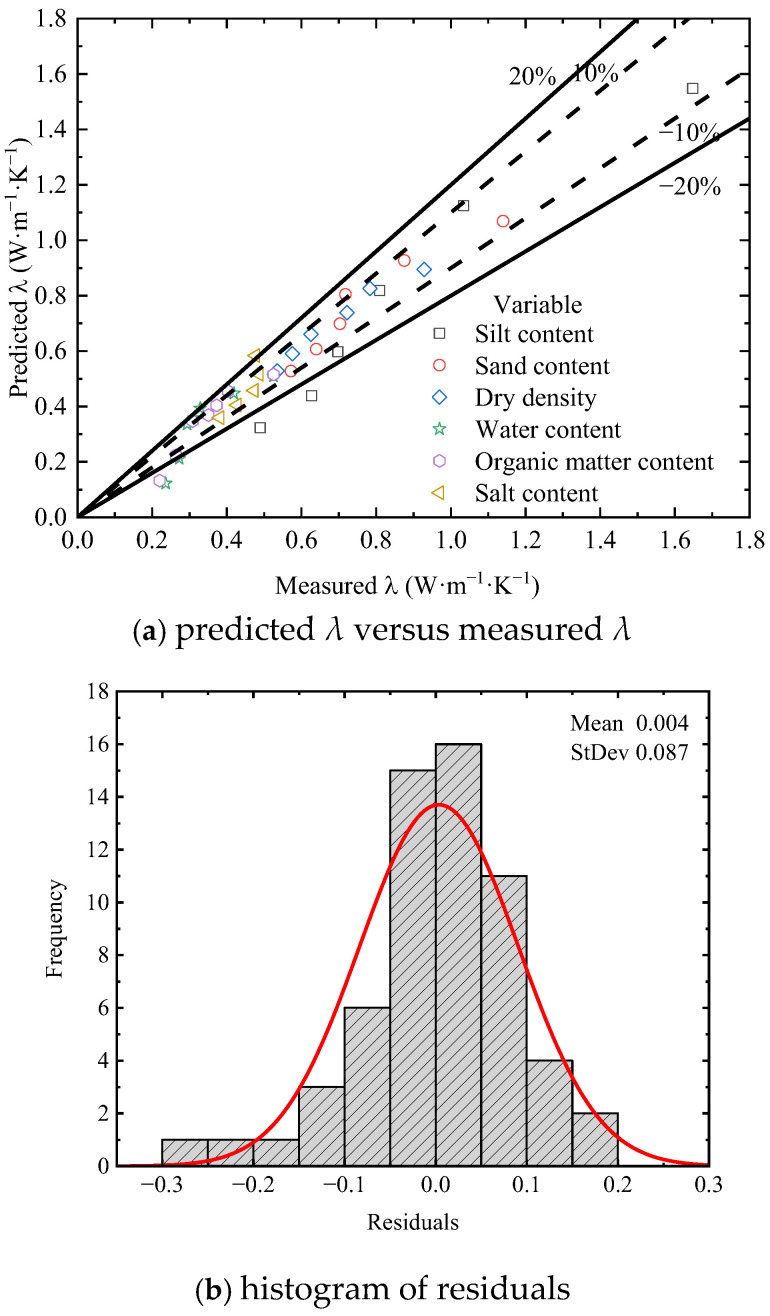
Error analysis of measured and predicted thermal conductivity of the modified model.

To assess the performance of the proposed modified model, an error analysis was conducted as shown in Figure 10. Figure 10a presents a scatter plot comparing measured *λ* and predicted *λ* by the modified model. Most data points cluster near the 1:1 line, suggesting that the modified model performs reasonably well. Few data points, mostly from the silt content, are outside the ±20% error bands. Figure 10b displays the histogram of residuals, i.e., the differences between measured and predicted *λ* values. The residuals are predominantly distributed within a narrow range around zero, showing an approximate symmetric pattern. This indicates that the model errors are random and unbiased, without systematic overestimation or underestimation. By calculating the root mean squared error (RMSE) and coefficient of determination (R^2^), the proposed modified model decreases the RMSE from 0.295 to 0.087 and increases the R^2^ from −0.55 to 0.866, thereby significantly enhancing the predictive performance of the model.

## 5. Conclusions

This study systematically explores the thermal conductivity characteristics of silty clay by the AHFO-FBG technology. The primary conclusions are drawn as follows:

(1) Influence mechanisms of particle content, dry density, moisture content, organic matter content, and salt content on thermal conductivity were investigated. The thermal conductivity of silty clay decreases monotonically with increasing silt or sand content, with silt content exerting a more significant impact than sand content. Thermal conductivity increases with the rise in dry density. Thermal conductivity exhibits a “first increasing then decreasing” trend with varying moisture content and salt content, with critical thresholds at approximately 50% and 2%, respectively. Thermal conductivity decreases with the increase in organic matter content, with a more pronounced reduction observed at low organic matter contents. The increase in potassium humate content reduces the thermal conductivity of soil slightly more than sodium humate.
(2) Based on the sensitivity analysis under reference values, the sensitivity ranking of factors affecting soil thermal conductivity is: dry density > sodium humate > silt content > potassium humate > moisture content > sand content > salt content. This indicates that dry density is the most sensitive factor to thermal conductivity changes. It is necessary to minimize the measurement error of dry density by increasing test repetitions, and data collection should retain maximum decimal places.
(3) A modified thermal conductivity prediction model is proposed by improving the Johansen (1977) model [23]. This model introduces shape factors that are linearly correlated with particle content, dry density, moisture content, organic matter content, and salt content, optimizing the relationship between normalized thermal conductivity and degree of saturation. Validation results show that the modified model significantly enhances predictive performance.

This study provides an accurate method for measuring thermal conductivity and enhances understanding of the heat-transfer mechanism in silty clay. However, practical engineering applications require deeper exploration of the sensor’s long-term reliability and environmental adaptability—such as its performance and package integrity under repeated heating–cooling cycles and the corrosion risk of its materials.

## Figures and Tables

**Table 1:** Properties of tested soil.

Liquid Limit	Plastic Limit	Organic Matter Content	Maximum Dry Density	Optimum Moisture Content
40%	25%	7.98%	1.57 g/cm^3^	20%

**Table 2:** Technical parameters of the demodulator.

Number of Channels	Wavelength Coverage /nm	Wavelength Resolution/pm	Reproducibility /pm	Demodulation Rate /Hz	Type of Optical Cable Port	Electric Power Supply
16	1527~1568	1	±3	≤1	FC/APC	+12 V/2 A

**Table 3:** Experimental scheme.

Variable	Variation Range	Reference Value
Silt content (%)	10, 20, 30, 40, 50, 60	37
Sand content (%)	10, 20, 30, 40, 50, 60	44
Dry density (g/cm^3^)	1.1, 1.2, 1.3, 1.4, 1.5, 1.6	1.3
Water content (%)	10–155	20
Organic matter content (%)	8, 12, 16, 19, 21, 36, 55	8
Salt content (%)	1, 2, 3, 4, 5	1

Note: The dry density of the soil was controlled when the moisture content was less than 40%. The organic matter and salt contents represent mass percentages of the substances added to the soil.

**Table 4:** Sensitivity factors of the influencing factors.

Factors	System Characteristic *P*, i.e., Thermal Conductivity *λ*	Sensitivity Factor S*(X*)	Reference Value X*	S* Value	Rank
Silt	$\lambda_{silt}=7.456X_{silt}^{-0.670}+0.048$	$S_{siit}^{*}(X_{siit}^{*})=\left|-5.0\times {\left(X_{siit}^{*}\right)}^{-1.67}\right|X_{siit}^{*}/\lambda_{siit}^{*}$	37	0.625	3
Sand	$\lambda_{sand}=2.897X_{sand}^{-0.486}+0.193$	$S_{sand}^{*}(X_{sand}^{*})=\left|-1.408\times \left(X_{sand}^{*}\right)-1.486\right|X_{sand}^{*}/\lambda_{sand}^{*}$	44	0.342	6
Dry density	$\lambda_{\rho }=0.034X_{\rho }^{5.590}+0.480$	$S_{\rho }^{*}(X_{\rho }^{*})=\left|0.19\times {\left(X_{\rho }^{*}\right)}^{4.590}\right|X_{\rho }^{*}/\lambda_{\rho }^{*}$	1.3	1.313	1
Moisture content	$\lambda_{w}=\left\{\begin{array}{ll} -2.356X_{w}^{-0.231}+1.731 & X_{w}<50\%\;\\ -3.198\times 10^{-5}X_{w}^{1.851}+0.802 & X_{w}\geq 50\%\; \end{array}\right.$	$S_{w}^{*}(X_{w}^{*})=\left\{\begin{array}{ll} \left|0.544\times {\left(X_{w}^{*}\right)}^{-1.231}\right|X_{w}^{*}/\lambda_{w}^{*} & X_{w}^{*}\text{<}50\%\;\\ \left|-5.919\times 10^{-5}\times {\left(X_{w}^{*}\right)}^{0.851}\right|X_{w}^{*}/\lambda_{w}^{*} & X_{w}^{*}\geq 50\%\; \end{array}\right.$	20	0.494	5
Sodium humate	$\lambda_{org\_s}=4.872X_{org\_s}^{-1.318}+0.215$	$S_{org\_s}^{*}(X_{org\_s}^{*})=\left|-6.421\times {\left(X_{org\_s}^{*}\right)}^{-2.318}\right|X_{org\_s}^{*}/\lambda_{org\_s}^{*}$	8	0.783	2
Potassium humate	$\lambda_{org\_p}=1.818X_{org\_p}^{-0.714}+0.131$	$S_{org\_p}^{*}(X_{org\_p}^{*})=\left|-1.298\times {\left(X_{org\_p}^{*}\right)}^{-1.741}\right|X_{org\_p}^{*}/\lambda_{org\_p}^{*}$	8	0.512	4
Salt	$\lambda_{sait}=0.036X_{sait}-0.010X_{sait}^{2}+0.456$	$S_{sait}^{*}(X_{sait}^{*})=\left|0.36-0.20X_{sait}^{*}\right|X_{sait}^{*}/\lambda_{sait}^{*}$	1	0.033	7

## Data Availability

The datasets for this study are available from the corresponding author upon reasonable request.
